# Neural Bases of Age-Related Sensorimotor Slowing in the Upper and Lower Limbs

**DOI:** 10.3389/fnagi.2022.819576

**Published:** 2022-05-03

**Authors:** Uros Marusic, Manca Peskar, Kevin De Pauw, Nina Omejc, Gorazd Drevensek, Bojan Rojc, Rado Pisot, Voyko Kavcic

**Affiliations:** ^1^Science and Research Centre Koper, Institute for Kinesiology Research, Koper, Slovenia; ^2^Department of Health Sciences, Alma Mater Europaea – ECM, Maribor, Slovenia; ^3^Biological Psychology and Neuroergonomics, Department of Psychology and Ergonomics, Faculty V: Mechanical Engineering and Transport Systems, Technische Universität Berlin, Berlin, Germany; ^4^Human Physiology and Sports Physiotherapy Research Group, Vrije Universiteit Brussel, Brussels, Belgium; ^5^Brussels Human Robotics Research Center (BruBotics), Vrije Universiteit Brussel, Brussels, Belgium; ^6^Jožef Stefan Institute, Ljubljana, Slovenia; ^7^Jožef Stefan International Postgraduate School, Ljubljana, Slovenia; ^8^Faculty of Mathematics, Natural Sciences and Information Technologies, University of Primorska, Koper, Slovenia; ^9^Faculty of Medicine, University of Ljubljana, Ljubljana, Slovenia; ^10^Department of Neurology, Izola General Hospital, Izola, Slovenia; ^11^Institute of Gerontology, Wayne State University, Detroit, MI, United States

**Keywords:** aging, event-related potential (ERP), visual-evoked potential (VEP), motor-related potential, finger and foot responses

## Abstract

With advanced age, there is a loss of reaction speed that may contribute to an increased risk of tripping and falling. Avoiding falls and injuries requires awareness of the threat, followed by selection and execution of the appropriate motor response. Using event-related potentials (ERPs) and a simple visual reaction task (RT), the goal of our study was to distinguish sensory and motor processing in the upper- and lower-limbs while attempting to uncover the main cause of age-related behavioral slowing. Strength (amplitudes) as well as timing and speed (latencies) of various stages of stimulus- and motor-related processing were analyzed in 48 healthy individuals (young adults, *n* = 24, mean age = 34 years; older adults, *n* = 24, mean age = 67 years). The behavioral results showed a significant age-related slowing, where the younger compared to older adults exhibited shorter RTs for the upper- (222 vs. 255 ms; *p* = 0.006, respectively) and the lower limb (257 vs. 274 ms; *p* = 0.048, respectively) as well as lower variability in both modalities (*p* = 0.001). Using ERP indices, age-related slowing of visual stimulus processing was characterized by overall larger amplitudes with delayed latencies of endogenous potentials in older compared with younger adults. While no differences were found in the P1 component, the later components of recorded potentials for visual stimuli processing were most affected by age. This was characterized by increased N1 and P2 amplitudes and delayed P2 latencies in both upper and lower extremities. The analysis of motor-related cortical potentials (MRCPs) revealed stronger MRCP amplitude for upper- and a non-significant trend for lower limbs in older adults. The MRCP amplitude was smaller and peaked closer to the actual motor response for the upper- than for the lower limb in both age groups. There were longer MRCP onset latencies for lower- compared to upper-limb in younger adults, and a non-significant trend was seen in older adults. Multiple regression analyses showed that the onset of the MRCP peak consistently predicted reaction time across both age groups and limbs tested. However, MRCP rise time and P2 latency were also significant predictors of simple reaction time, but only in older adults and only for the upper limbs. Our study suggests that motor cortical processes contribute most strongly to the slowing of simple reaction time in advanced age. However, late-stage cortical processing related to sensory stimuli also appears to play a role in upper limb responses in the elderly. This process most likely reflects less efficient recruitment of neuronal resources required for the upper and lower extremity response task in older adults.

## Introduction

To avoid falls and injuries in our environment, we must perceive the threat and then select and execute the appropriate response. Bypassing unprecedented external perturbations in one’s immediate proximity, such as stepping over an uneven surface and maintaining balance, requires sensory sharpness and fast reactions to the perceived sensory stimulus (Stelmach and Worringham, 1985). Loss of sensory acuity (Cavazzana et al., 2018) and reaction speed (Deary and Der, 2005) associated with advancing age may contribute to factors like increased risks of falls and trips. For example, older fallers are more likely to suffer from impaired sensory acuity than older non-fallers (Lord et al., 1991; Brundle et al., 2015). Similarly, evidence obtained from reaction time (RT) tasks in older adults have demonstrated that increased RT of finger pressing is a significant and independent risk factor for falls (Lord et al., 1991, 1994; Lord and Clark, 1996). From the perspective of avoiding danger, such as rebalancing when confronted with an obstacle or slipping, rapid foot movements to maintain balance play an essential role (Pijnappels et al., 2010; Cai et al., 2020) which might be even more important than the upper-limb movement reactions. In a standardized clinical testing protocol, lower-limb RTs to visual stimuli discriminated between single and multiple fallers, providing a simple, cost- and time-effective way of identifying people at greatest risk of falling (Maver et al., 2011). However, the investigation of simple RT of the lower limbs remains a severely under investigated area. This study aimed to fill this gap and provide the first insights into the origin of sensorimotor delay in the upper- *and* lower limbs using electroencephalography (EEG).

Age-related behavioral slowing is typically reflected in longer RTs to simple auditory and visual stimuli (Woodruff and Kramer, 1979; Gottsdanker, 1982; Fozard et al., 1994; Inui, 1997; Deary and Der, 2005), and even more so in the tasks of higher-level cognition, such as sustained and selective attention, inhibitory control, working memory, and executive control (Finnigan et al., 2011; Gajewski and Falkenstein, 2014; Reuter et al., 2019). In a simple RT task, Woods et al. (2015) showed a 0.55-millisecond increase per year in mean simple RT in a visual paradigm, whereas Gottsdanker (1982) showed a 2-millisecond increase per decade in an auditory paradigm. In a more challenging GO/NOGO paradigm, Kropotov et al. (2016) demonstrated that early perceptual components (100–200 milliseconds) of event-related potentials (ERP) increased their peak latencies for 5–6 milliseconds per decade, while the later components (400–500 milliseconds) showed approximately 16 milliseconds increase pre decade. The structural damage to the myelin sheath and the reduction of the total number of nerve fibers in aged individuals (Salat, 2011), might undermine the functional efficacy of information flow by disrupting precisely timed communication patterns or rhythmic synchronization among cortical regions (Fries, 2015). Age-related behavioral slowing or motor impairments are not limited to associated changes in white matter structure (demyelization), but also manifest due to changes in gray matter (reduced volume, atrophy), biochemical effects (including reduced dopamine levels, receptors, transmission and transporters) and functional neural recruitment (for a review, see Seidler et al., 2010).

Simple RT, a basic measure of the minimal time required to respond to a stimulus, provides an estimate of the overall alertness and motor speed and is highly dependent on sensorimotor integration (Azim and Kazuhiko, 2019). Responses in a simple RT task can be therefore broken into a sensory processing stage in which a stimulus is perceived or detected, followed by a motor processing stage, during which the necessary movement is prepared and executed as a response (Woods et al., 2015). Despite being primarily used to assess processing speed, the RT task is nevertheless a cognitive-based test as attentional resources are needed not only for stimulus detection but also for movement initiation (Cai et al., 2020). Recorded reaction times comprise a summed duration of these events. Given that sensory and motor processing stages are functionally different, determining the basis of the behavioral slowing is an important step for an adequate understanding of aging-related changes and for intervention development. Attempts of estimating and dividing the stimulus detection time from movement initiation time were done on a behavioral level and suggest that the age-related sensorimotor slowing occurs primarily due to slowed motor output rather than the stimulus detection time (Woods et al., 2015). However, the characteristics of sensory- and motor-related processing stages of RTs investigated from the angle of their respective neurodynamic signatures remain spared for the upper extremities and have not yet been studied in the lower extremities.

Electroencephalography offers a way to investigate the origins of age-related sensorimotor delay by analyzing the time course of internal responses to sensorimotor information processing in conjunction with RT tasks. In the stimulus processing stage, the recognition of the stimulus occurs, which is captured by the early stimulus-locked ERP (s-ERP) components – P1 and N1 (Gazzaniga et al., 2019). The components’ latencies indicate speed, while their amplitudes indicate the intensity of early perceptual mechanisms (Amenedo and Díaz, 1998). Visually evoked P1 peak is typically detected within the 40–140 millisecond range after the stimulus presentation, while the N1 is detected within the 120–200 millisecond range (Yordanova et al., 2004; Zalar et al., 2015; Gazzaniga et al., 2019). P1 and N1 are proposed to reflect gain control of the sensory processing (Luck et al., 1990; Klimesch et al., 2004, 2007), and can be modulated by attention (Gazzaley et al., 2008; Gazzaniga et al., 2019). In the visual paradigm, the P1 and N1 components are observed and generated in the extrastriate cortex (Natale et al., 2006). Later component – P2, typically peaks at/after 200 milliseconds after stimulus presentation and is evidently generated in parieto-occipital regions, rostral to extrastriate cortex (Freunberger et al., 2007). P2 component has primarily been associated with higher-level cognitive functions, such as working memory (Wolach and Pratt, 2001; Lefebvre et al., 2005), encoding (Dunn et al., 1998) and semantic processing (Freunberger et al., 2007), and is therefore not surprising that its generating source corresponds to the parieto-occipital association cortex.

Motor processing can be examined by the motor-related cortical potentials (MRCPs), which are computed by averaging response-locked ERPs (r-ERPs) at the contralateral motor cortical sites (Taniguchi et al., 2001). This allows for the analysis of the latency and amplitude of the most negative MRCP peak and its comparison between older and younger adults. Yordanova et al. (2004) report no differences in the peak MRCP amplitude between the older and younger adults on the contralateral side to the responding hand, however, significantly larger ipsilateral activity detected in older adults might be indicative of deviant functional asymmetry and functional dysregulation of the motor cortex activation in older adults (Mattay et al., 2002; Ward and Frackowiak, 2003; Heuninckx et al., 2005, 2008). Despite the absence of evidence of behavioral RT slowing in older compared to younger adults in Yordanova et al. (2004), their findings are indicative of age-related neural deterioration. It remains unclear, however, whether the generally observed slowing of simple reaction times in older adults originates from the perceptual processing stage or motor-related processing stage. Evidence obtained in a task with higher complexity (choice RT) indicated amplitude increase and prolongation of the MRCP contralateral to the responding hand. This was not due to changes in the sensory stimulus processing phase, as observed in the latency and amplitude of the early ERP components, or in the response selection phase, which was investigated by the onset of the lateralized readiness potential (LRP) (Yordanova et al., 2004; Falkenstein et al., 2006).

Taken together, the evidence shows that the sensorimotor slowing observed in RT tasks in aged individuals may be primarily due to alterations of motor-related processing rather that stimulus detection processing. However, this statement is not strongly supported in the context of the simple RT task. In addition, the electrophysiological underpinnings of the lower-limb simple RT performance have never been investigated before. It remains unclear how the typically observed age-related sensorimotor slowing in the context of simple RT tasks relates to the associated neurodynamics at the level of stimulus detection and motor processing stages. In the present study, we investigate the simple RTs of the upper- and lower extremities in younger and older adults to assess the effects of aging on processing speed. Therefore, the aim is to investigate the origin of age-related behavioral sensorimotor slowing on a neural level for the upper limb and, for the first time, in the lower limb.

## Materials and Methods

### Study Design and Participants

This cross-sectional study was conducted with a sample of 48 healthy adults, half of whom were younger (*N* = 24; mean age = 34 years; 11 men) and the other half were older adults (*N* = 24; mean age = 67 years; 9 men). All procedures were carried out in accordance with the ethical standards of the 1964 Declaration of Helsinki and were approved by the National Medical Ethics Committee (No. KME 57/06/17). Written informed consent was obtained from all participants prior to study enrollment.

Table 1 represents the basic characteristics of both groups. The younger adults were higher educated compared to older adults (16 years vs. 13 years, respectively, *p* < 0.001). Also, younger adults were taller compared to older adults (176 cm vs. 166 cm, respectively, *p* = 0.010). All participants stated that they were satisfied with their current health status, and no older adults reported an incidence of cardiovascular or neurological disease or took medication against it. Four young adults reported a left-hand preference, and all older adults reported a right-hand preference. All participants reported normal or corrected to normal vision and were able to clearly understand and follow the instructions of the simple RT task with upper- and lower limbs.

**TABLE 1:** Table of basic characteristics of young and older adults.

Variables	Young adults	Older adults	*p* value
N	24	24	
Sex (m/f)	11/13	9/15	
Age (years)	34.1 ± 2.3	66.8 ± 4.4	<0.001
BMI (kg/m^2^)	24.0 ± 2.1	26.9 ± 6.2	0.206
Education (years)	16.4 ± 2.0	13.4 ± 1.8	<0.001
MoCA score (0–30)		27.5 ± 1.6	
TMT-A (sec)	24.7 ± 5.5	41.5 ± 18.2	<0.001
TMT-B (sec)	40.0 ± 26.9	76.5 ± 26.3	<0.001

*BMI, body mass index; MoCA, Montreal Cognitive Assessment; TMT, trail-making test.*

### Neuropsychological Assessment

The *Montreal Cognitive Assessment* (MoCA) was used to obtain a general level of cognitive performance and to test for cognitive impairment only in the sample of older adults (Nasreddine et al., 2005). The MoCA test refers to several cognitive domains, namely visual-spatial abilities, short-term memory, executive functions, attention, concentration, working memory, language, and temporal and spatial orientation. The final score ranges from 0 to 30 points, with scores ≥ 26 indicating no cognitive impairment.

The *Trail-Making Test* (TMT; Reitan, 1958; Tombaugh, 2004) was used to assess the speed of processing and executive function. In TMT-A, 25 encircled numbers randomly distributed on an A4 paper format must be connected with a single line in ascending order, starting at 1 and ending at 25. In TMT-B, a line must be drawn connecting numbers (1 – 12) and letters (A – L) in an alternating and increasing fashion (1-A-2-B-3-C…12-L). The scores for each part are given in time to completion (in seconds).

### A Simple Visual Reaction Task

**FIGURE 1:**
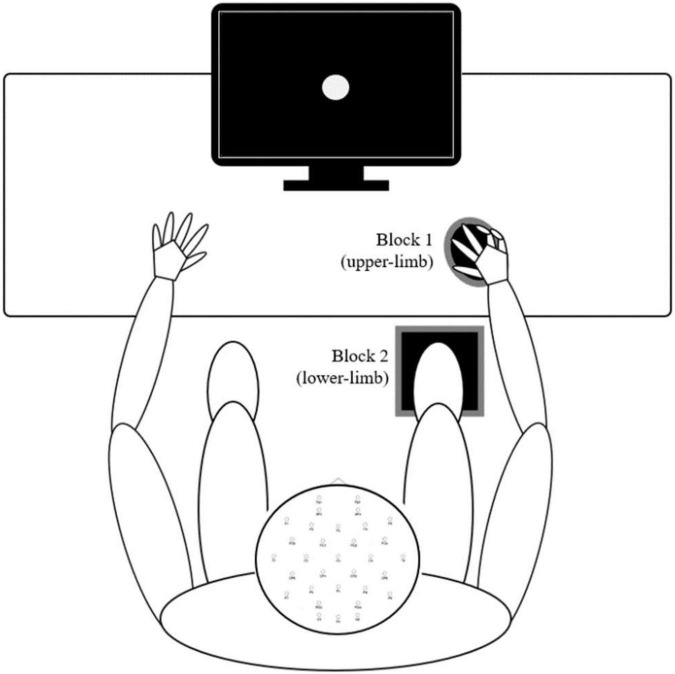
Experimental procedures – Participants sat in a natural posture on a comfortable chair while maintaining visual fixation in the center of the screen in front of them. The two conditions (upper- and lower-limb) were applied in separate blocks, and the order of these blocks was randomized. In both conditions, the finger and foot rested on the response pad between responses.

A simple visual reaction task (also called the psychomotor vigilance task) is a sustained attention reaction-timed performance task that measures the speed with which participants respond to a visual stimulus (Dinges and Powell, 1985). The experimental setup is presented in Figure 1. Participants were assessed while seated in a neutral position and instructed to perform a simple reaction time test in response to 70 visual stimuli presented with a random interstimulus interval between 2 and 5 s on a 17.0-inch flat panel LCD monitor (120-Hz refresh rate) situated approximately 50 centimeters in front of them. Their task was to press the response button as quickly as possible with (a) their index finger of their dominant hand and (b) the bottom of the right foot (second metatarsal head). The two conditions were applied in separate blocks (upper- and lower-limb). The order of these blocks was randomized. In both cases, the finger and foot rested on the response pad between responses. The response pad was connected to a trigger box (g.tec TRIGbox). The visual stimuli were presented in the center of the monitor (circular disc with a 5 cm radius was presented against a black background at the center of the display, duration 150 ms, intensity 50 cd/m2, visual angles 1° horizontal/1.5° vertical) placed directly in front of the participant’s visual field. Additional visual stimuli (not visible to the participant) were simultaneously presented with experimental stimuli and were recorded using photodiodes connected directly to the trigger box – a methodology that afforded precise stimulus onset and offset trigger times that were subsequently embedded within each participants raw EEG data file. RTs were then extracted from an event/marker list for each subject using a script written for the MATLAB software (The MathWorks, Inc., Natick, MA, United States, version MATLAB R2021a). Trials with RTs latencies less than 110 ms and greater than 1,000 ms were excluded as outliers (Woods et al., 2015). Based on this criterion, up to four and seven trials were discarded in a group of young and older adults, respectively.

### Electroencephalography Recording and Analysis

Scalp EEG activity was recorded using g.tec medical engineering equipment (Schiedlberg, Austria), with 32 Ag/AgCl active electrodes, arranged according to the International 10–20 System. Included electrodes correspond to Fp1, Fp2, AF3, AF4, F7, F3, Fz, F4, F8, FC5, FC1, FC2, FC6, T7, C3, Cz, C4, T8, CP5, CP1, CP2, CP6, P7, P3, Pz, P4, P8, PO3, PO4, O1, Oz, and O2 predefined positions. The reference electrode was placed on the left earlobe and the ground electrode was placed in the AFz position. During EEG measurements, low-pass and high-pass filters were set to 100 and 0.1 Hz, respectively, for real-time display only. The notch filter was set to 50 Hz. Impedances were maintained below 10 kΩ for each channel and balanced across all channels within a 5 kΩ range. The sampling rate was 512 Hz with a 32-bit resolution. Before performing the simple visual reaction task, a baseline measurement was routinely performed with eyes open and eyes closed (3 min each) to check the quality of the EEG signal.

All data were preprocessed and analyzed using custom scripts in the EEGLAB toolbox (Delorme and Makeig, 2004) of the MATLAB software (The MathWorks, Inc., Natick, MA, United States, version MATLAB R2021a). First, the upper- and lower-limb recordings of single participants were concatenated into a single data file, down sampled to 256 Hz, high-pass filtered at 1 Hz. Second, a copy of the original concatenated EEG data file (copyEEG) was created. The copyEEG was average re-referenced, automatic bad channel detection algorithm *clean_artifacts* was applied (*FlatLineCriterion* was set to 10 s, *ChannelCriterion* to 0.80, and *ChannelCriterionMaxBadTime* to 0.5) and the bad channels detected were interpolated using the spherical method. Next, the CopyEEG was epoched to stimulus-locked intervals [−200 800 ms], and an inspection of rejected and accepted epochs was made using the amplitude threshold of >100 μV. This intermediate inspection determined if the automated bad channel detection and interpolation sufficiently cleaned the data or more channels had to be interpolated as a tradeoff to preserve the highest possible number of epochs and consequently grant the highest quality of the ERP signal. If a channel (i) was exceeding the 100 μV on a significant number of epochs and (ii) was not of primary interest to our analyses (other than occipital and central electrode sites) and (iii) was not detected by the automatic procedure, it was manually rejected and interpolated at this stage. The labels of the automatically and manually interpolated channels were saved into a variable and CopyEEG was discarded. The rationale for using CopyEEG was to first discover bad channels to then be able to interpolate them in the original concatenated EEG dataset *before* re-referencing it to the average reference. This approach offers a way to restrict the bad channel information from contributing to the average re-reference as it represents the non-meaningful information we are aiming to delineate from the signal of interest and reject.

In the original EEG dataset, bad channels were interpolated, and the data were re-referenced to the average reference. Time-domain cleaning eliminated data segments characterized by muscle artifacts, electrode pops, and other major perturbations. Next, the Adaptive Mixture Independent Component Analysis (AMICA) was performed using 2,000 iterations and subject-specific reduction of the data rank was considered (the number of interpolated channels plus one accounting for average re-referencing). The DIPFIT plugin for source localization was used to estimate single equivalent dipoles, co-registering the channel locations to *standard_BEM* head models. Lastly, the ICLabel plugin (Pion-Tonachini et al., 2019) was used to label the independent components. This procedure delivered a single independent component solution per subject and the concatenated data file was not used for further analyses.

In the final stage of the preprocessing, we returned to the separate condition data files (upper and lower limb files) which were treated in the same manner as described above (down sampling to 256 Hz, high pass filtering at 1 Hz, automatic and manual bad channel interpolation, and average re-referencing). Next, subject-specific AMICA (*icaweights*, *icasphere, icaact, icawinv, icachansind*) and DIPFIT information were copied to the subject’s separate condition data files and ICLabel was used. Independent components labeled as “eye” were automatically rejected if they met the threshold of ≥ 85%. Components expressing clear horizontal or vertical eye-movement profiles based on topography, spectral plot, and time-domain signature, but failed to reach 85% eye labeling threshold, were rejected manually.

For each experimental condition, the ERP analyses were performed on a single subject level. Stimulus-locked event-related potentials (s-ERPs) were segmented to −200 to + 800 ms epochs with a baseline correction set to −200 to 0 milliseconds. Epochs were rejected using the amplitude cutoff value of 100 μV (Yordanova et al., 2004). In all cases, a minimum of 50 stimuli epochs were averaged. ERPs to visual stimulation were assessed over the occipital locations where responses were most strongly represented (the occipital electrodes O1, Oz, and O2). The following peak-detection analyses were performed: (i) P1 was detected as the most positive peak (amplitude and latency) within the range 40–140 milliseconds after the stimuli occurred, (ii) N1 was the most negative peak (amplitude and latency) within the range of 80–140 milliseconds, and (iii) P2 was the first positive peak (amplitude and latency) after 200 milliseconds (Yordanova et al., 2004).

Additionally, the response-locked ERPs (r-ERP; for the upper- and lower-limb) were segmented to −500 to + 500 milliseconds epochs with the baseline set to −500 to −300 milliseconds before the response occurred. The exact methodological procedures implemented for the s-ERP epoch extraction (see above) were also utilized for the r-ERP extraction. r-ERPs, or motor-related cortical potentials (MRCPs) for simple reaction times, were analyzed over the C3 or C4 electrode, which was positioned on the contralateral side of the dominant hand above the motor cortex as well as above the Cz electrode, which overlays the sensorimotor cortices on the homunculus. The following parameters were extracted from each MRCP: the most negative displacement of MRCP (peak latency and amplitude), the onset latency of the MRCP with a threshold of 15% of MRCP maximum peak, and the duration of the motor-related activation, known as MRCP rise time (Yordanova et al., 2004). Because we focused on the pre-motor response potentials, we did not statistically evaluate the post-response potentials [the movement-monitoring potential known to be a component of performance control (Shakeel et al., 2015)], but we presented them in ERP and topographic figures for display purposes.

### Statistical Analyses

The mean reaction time and its within-subject variability [standard deviation (SD) across trials] were computed and analyzed with a custom script written for MATLAB software (MathWorks, Natick, MA, United States). The behavioral and ERP results were statistically processed in the SPSS software version 26.0 (IBM, Chicago, IL, United States). Homogeneity of variances and normality of distribution of parameters was tested using Levene’s test and Shapiro-Wilk test, respectively. A repeated-measures analysis of variance (RM ANOVA) with a within-subject factor of condition (upper- and lower-limb) and between-subject factor of age (young and older adults) was used to assess the main effects (age and condition) and the two-way interaction effect between age and condition. For significant effects, effect size as partial *η^2^* was reported and Bonferroni *post hoc* tests were applied. This included *post hoc* analysis for each significant main effect, primarily to present the mean differences and statistics related to the two levels in more detail. Multiple stepwise regression analysis was performed separately for age (young and older adults) and limb condition (upper- and lower-limb) to identify significant surviving predictors of RTs. Four models were run in which all ERP variables in each category (younger, older adults, upper- and lower-limb) were entered to predict RTs. Statistical conclusions were drawn at a *p*-value of 0.05.

## Results

### Behavioral Data

**FIGURE 2:**
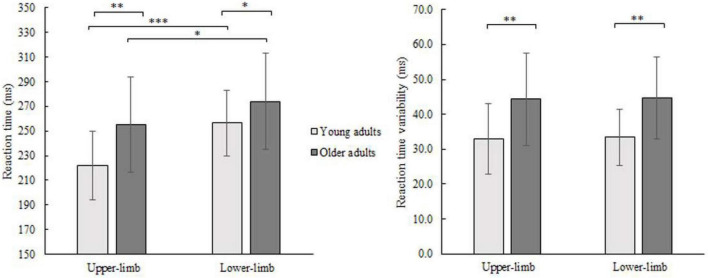
Mean RT (left) and its variability (SD) (right) of younger and older adults for lower- and upper-limb conditions. Error bars reflect one SD. * represents *p* < 0.05; ^**^ represents *p* < 0.01; ^***^ represents *p* < 0.001.

Average finger and foot RTs for younger and older adults are presented in Figure 2. The RM ANOVA showed that each of the main effects were significant, with age- [*F*(1,46) = 6.452, *p* = 0.008, Partial *η^2^* = 0.126], and condition-related differences [*F*(1,46) = 43.118, *p* < 0.001, Partial *η^2^* = 0.496]. The interaction was not significant (*p* = 0.110). Both groups had longer RTs responding with lower- compared to the upper-limb (older *p* = 0.038; younger *p* < 0.001) as well as older adults had longer RTs responding with upper- (*p* = 0.006) and lower-limb (*p* = 0.048) compared to their younger counterparts.

The variability of RTs (SD) showed a significant age effect [*F*(1,46) = 16.687, *p* < 0.001, Partial *η^2^* = 0.294] and no condition (*p* = 0.371) or interaction effect (*p* = 0.975). There was greater variability in older compared to younger adults while responding with the upper- (*p* = 0.001) as well as with lower limbs (*p* = 0.001).

### Electrophysiological Data

**FIGURE 3:**
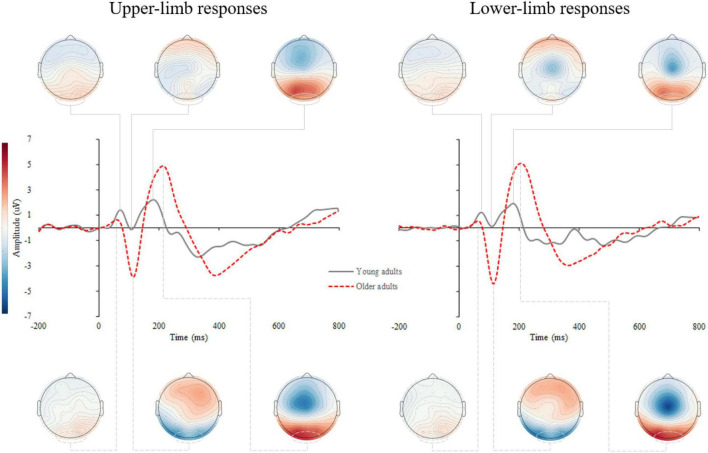
Grand average stimulus-locked event-related potentials (s-ERPs) for upper- (left) and lower-limb RT (right) for younger and older adults. The topographic maps correspond to the individual ERP components: P1, N1, and P2.

Supplementary Figure 1 shows the stimulus-locked event-related potentials (s-ERPs) on the scalp. An average s-ERP above the occipital cortex (electrode O1, Oz, and O2) is shown in Figure 3 with topographic patterns of P1, N1, and P2 components. Processing of visual stimuli was further investigated over the occipital electrodes and shows overall larger amplitudes with delayed latencies of endogenous potentials in older compared to younger adults.

### P1 Amplitude and Latency

The RM ANOVA for P1 amplitude did not show a significant effect of age (*p* = 0.141), condition (*p* = 0.374), or interaction (*p* = 0.432). Similarly, age (*p* = 0.337), condition (*p* = 0.271), and interaction (*p* = 0.263) effects were not significant for the P1 latency.

### N1 Amplitude and Latency

**FIGURE 4:**
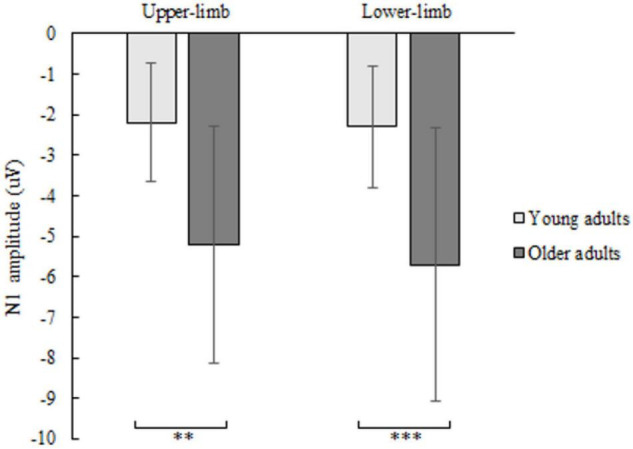
N1 amplitude of younger and older adults for lower- and upper-limb conditions. Error bars reflect one SD. * represents *p* < 0.05; ^**^ represents *p* < 0.01; ^***^ represents *p* < 0.001.

The N1 amplitude showed a significant age [*F*(1,46) = 12.160, *p* = 0.001, Partial *η^2^* = 0.311], but no condition (*p* = 0.404) nor interaction effect (*p* = 0.426). Older compared to younger adults had greater negative N1 deflection while responding with upper- (*p* = 0.001) as well as with lower limbs (*p* < 0.001) (Figure 4). No significant effect of age (*p* = 0.179), condition (*p* = 0.486) and interaction (*p* = 0.719) was found for N1 latency.

### P2 Amplitude and Latency

**FIGURE 5:**
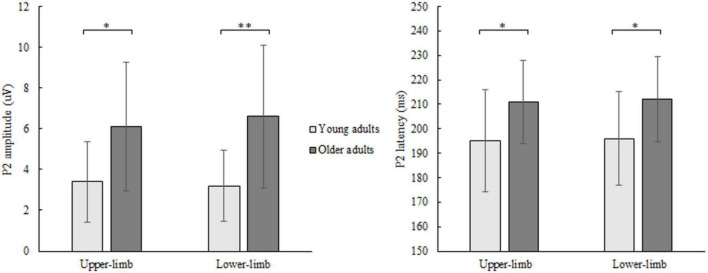
P2 amplitude (left) and latency (right) of younger and older adults for lower- and upper-limb conditions. Error bars reflect one SD. * represents *p* < 0.05; ^**^ represents *p* < 0.01; ^***^ represents *p* < 0.001.

The P2 amplitude showed a significant age [*F*(1,46) = 10.252, *p* = 0.003, Partial *η^2^* = 0.249], but no condition (*p* = 0.706) nor interaction effect (*p* = 0.133). Older compared to younger adults had greater P2 amplitude (Figure 5) while responding with both upper- (*p* = 0.011) and lower limbs (*p* = 0.001).

Similarly, for P2 latency a significant age [*F*(1,46) = 8.122, *p* = 0.011, Partial *η^2^* = 0.208], but no condition (*p* = 0.472) nor interaction effect (*p* = 0.577) was discovered. Prolonged P2 latency (Figure 5) was found in older compared to younger adults for both upper- (*p* = 0.011) and lower limbs (*p* = 0.024).

### Motor-Related Cortical Potential

**FIGURE 6:**
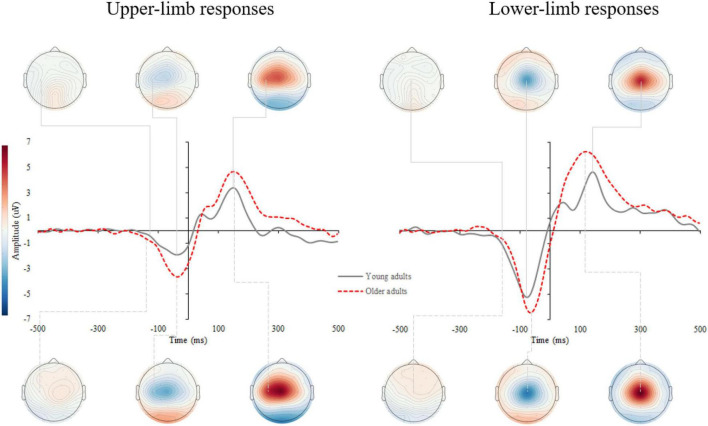
Grand average response-locked event-related potentials (r-ERPs) for upper- (left) and lower-limb RT (right) for younger and older adults. The topographic maps correspond to the individual MRCP components: onset latency, peak amplitude, and movement-monitoring potential.

Supplementary Figure 2 shows the response-locked event-related potentials (r-ERPs) on the scalp. Motor response processing was further examined over the contralateral motor cortex for upper-limb responses (C3 and C4 electrodes for the right- and left-handed participants, respectively) and at the vertex (Cz) for lower- limb responses. A grand average of the r-ERP is shown in Figure 6 with the topographic patterns of onset latency, MRCP peak, and movement-monitoring potential.

**FIGURE 7:**
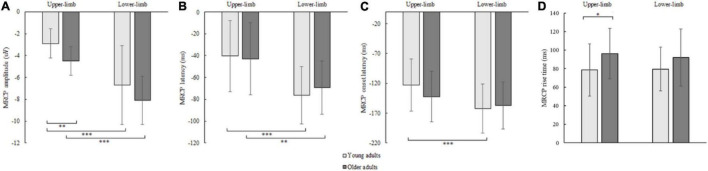
MRCP peak amplitude **(A)**, peak latency **(B)**, onset latency **(C)**, and rise time **(D)** of younger and older adults for lower- and upper-limb conditions. Error bars reflect one SD. * represents *p* < 0.05; ^**^ represents *p* < 0.01; ^***^ represents *p* < 0.001.

The RM ANOVA for MRCP peak amplitude showed an effect of age [*F*(1,42) = 4.914, *p* = 0.030, Partial *η^2^* = 0.117] and condition [*F*(1,42) = 16.446, *p* < 0.001, Partial *η^2^* = 0.586], while the interaction effect was not significant (*p* = 0.868). A larger MRCP peak amplitude was found in older compared to younger adults for upper- (*p* = 0.002) and a non-significant trend for lower limbs (*p* = 0.097). Although assessed from a different position (upper-limb at C3 or C4, and lower limb at Cz location), the MRCP peak amplitude was larger for lower- compared to upper-limb in younger (*p* < 0.001) and older adults (*p* < 0.001) (Figure 7A).

The MRCP peak latency showed a significant condition [*F*(1,42) = 62.855, *p* < 0.001, Partial *η^2^* = 0.605] but no age (*p* = 0.646) or interaction effect (*p* = 0.132). The MRCP peak latency occurred earlier for lower- compared to upper-limb in younger (*p* < 0.001) and older adults (*p* = 0.004) (Figure 7B).

The MRCP onset latency showed a significant condition effect [*F*(1,42) = 26.646, *p* < 0.001, Partial *η^2^* = 0.477], but no age (*p* = 0.624) or interaction effect (*p* = 0.116). Longer MRCP onset latencies were found for lower- as compared to upper-limb in younger (*p* < 0.001) and only non-significant trend in older adults (*p* = 0.095) (Figure 7C).

The MRCP rise time showed a significant age effect [*F*(1,42) = 4.242, *p* = 0.046, Partial *η^2^* = 0.192], but no condition (*p* = 0.683) or interaction effect (*p* = 0.526). Longer MRCP rise times were found in older compared to younger adults for the upper limb (*p* = 0.036), and a non-significant trend for the lower-limb (*p* = 0.072) (Figure 7D).

### Regression Analyses

A multiple stepwise regression analysis (Table 2) revealed that in young adults, upper-limb RTs were significantly predicted by MRCP onset latency [*F*(1,22) = 8.398, *R*^2^ = 0.39, *p* = 0.017], while in older adults, the upper-limb RTs were significantly predicted by three parameters, i.e., MRCP onset latency, MRCP rise time, and P2 latency [*F*(3,19) = 26.068, *R*^2^ = 0.89, *p* < 0.001]. Lower-limb RTs were significantly predicted by MRCP onset latency parameter in both younger [*F*(1,22) = 9.668, *R*^2^ = 0.40, *p* = 0.009] and older adults [*F*(1,19) = 4.275, *R*^2^ = 0.25, *p* = 0.043].

**TABLE 2:** Results of multiple regression analysis with surviving predictors presented.

Model	*R*	*R* ^2^	*B*	Std error	Beta	*p* value
**Upper-limb – Young adults**						
MRCP onset latency	0.625	0.391	−0.373	0.135	−0.625	0.017
**Upper-limb – Older adults**						
MRCP onset latency	0.762	0.580	−1.025	0.124	−1.022	<0.001
MRCP rise time	0.891	0.794	−0.750	0.196	−0.475	0.002
P2 latency	0.943	0.890	0.811	0.242	0.325	0.005
**Lower-limb – Young adults**						
MRCP onset latency	0.630	0.397	−0.493	0.162	−0.630	0.009
**Lower-limb – Older adults**						
MRCP onset latency	0.496	0.246	−0.508	0.230	−0.496	0.043

*All ERP components were entered into a stepwise regression model to predict RTs; R, multiple correlation coefficient; R^2^, adjusted coefficient of determination; B, regression coefficient; beta, standardized regression coefficient.*

## Discussion

This study aimed to investigate the age-related slowing of sensorimotor processes while reacting to visual stimuli with the upper and lower extremities. To this end, the EEG/ERP method was used to assess the strength and timing of different ERP components that reflect processing efficiency of the brain. In a sample of forty-eight healthy adults, we found an age-related slowing of simple RTs when responding with the upper- and lower- limbs. Variability was higher in older adults but consistent for both upper- and lower-limb performance. Further examination of s-ERP components revealed overall larger amplitudes with delayed latencies of endogenous potentials in older compared to younger adults. In addition, motor processes in older adults showed age-dependent deflections with higher MRCP amplitude, most likely reflecting less efficient recruitment of neuronal resources required for the execution of the sensorimotor task with the upper and lower limbs. Our study also suggests that specifically for the upper-limb RT in older adults, the P2 component plays an important role in addition to the MRCP parameters.

A general age-related slowing of behavior when responding to a simple visual reaction task was confirmed in our study. Most studies reported delayed upper-limb responses to simple visual stimuli in older compared to younger adults (Inui, 1997; Deary and Der, 2005; Ashoke et al., 2010), while fewer studies focused on lower-limb RTs (Lord and Fitzpatrick, 2001; Cai et al., 2020). In contrast, Yordanova et al. (2004) found no age-related slowing in upper-limb simple RT but confirmed it in choice RT while responding to visual stimuli. Increasing age was also expected to result in greater variability in RTs (for a review see Dykiert et al., 2012), however, inter-limb differences in each age group were not present, implying that all participants were able to maintain the same variability while responding with lower- and upper- limbs.

The origins of age-related behavioral slowing were further analyzed using ERP components reflecting stimulus-related processing. First, the results of our study showed no age-related changes in the early component of P1. Early perceptual processes addressed by the P1 (and also N1) component presumably reflect the gain control of sensory processing (Luck et al., 1990; Klimesch et al., 2004, 2007) and, according to previous reports, did not differ between young and old adults (Yordanova et al., 2004; Kavcic et al., 2013). However, other studies reported an age-related enhancement of early components in the processing of visual stimuli that follows the U-shape across the lifespan, with the amplitudes of P1 and N1 being larger in children and the elderly (Reuter et al., 2019). Second, greater negativity in N1 peak amplitude was found in older adults, but no difference in N1 latency. This enhancement (greater negativity) in N1 amplitude could indicate higher attentional resources recruited for the same amount of visual information processed (Hillyard and Anllo-Vento, 1998). If a simple RT task is considered more demanding in advanced age than in younger years, the processes involved in motor preparation and execution must be guided with a stronger reference to external stimuli, and for this reason, more attention is allocated to these stimuli in order to support movement execution (Yordanova et al., 2004). Third, enhanced and delayed P2 responses were observed in old compared with young adults, but there were no differences between the upper- and lower-limbs in any of the age groups. Previous studies examining P2 suggested that this component reflects an index of working memory (Lefebvre et al., 2005; Finnigan et al., 2011), stimulus salience (Riis et al., 2009), and stimulus evaluation (Potts, 2004). It has also been suggested that P2 plays an important role in top-down cognitive control (Karamacoska et al., 2019; Lai et al., 2020). Therefore, together with the significant differences between age groups in the Trail-Making Test (TMT) [indicative of visual scanning ability and working memory (Arbuthnott and Frank, 2020; Ciolek and Lee, 2020)], the age-related increase in P2 amplitude and prolonged P2 latency are indeed indicative of impaired cognitive control.

Here, the P2 latency was found to be a significant predictor of RTs, but only for older adults and the upper limbs. Why P2 latency had no predictive power for lower-limb performance RT, should be further investigated. The overall predictiveness of upper-limb RTs was also not consistent with Yordanova et al. (2004), who reported N1 latency (in addition to the MRCP components discussed below) as a significant predictor of simple RT in both young and older adults. Based on their results, we also hypothesized that N1 latency would be predictive at least for simple RT for upper-limb, however, we did not find any evidence of this. The discrepancy between our results and those of Yordanova et al. (2004) could be due to several factors, including differences in age structure (young 34 year vs. 23 year; older 67 year vs. 58 year, respectively). In the study by Reuter et al. (2019), the N1 component is shown to follow a u-shaped pattern, with the shortest latencies and smallest amplitudes occurring in middle-aged individuals.

The negative potential preceding the movement represents the brain activity that is processed during the planning and preparation of a voluntary movement (Shibasaki and Hallett, 2006). It has been suggested that additional neural resources must be recruited in old age for successful motor performance (Heuninckx et al., 2008), while Yordanova et al. (2004) suggested that greater MRCP deflection was indicative of more extensive depolarization of neurons of the contralateral motor cortex (Yordanova et al., 2004). Similarly, the results of our study showed an age-related stronger MRCP amplitude for upper- and a trend for lower limbs but (although assessed from different positions) the MRCP amplitude was larger for lower- compared to upper-limb in both age groups. The question of why there is less efficient recruitment of neuronal resources required for motor response in the aging brain should be further explored by bringing together different imaging techniques. The review by Seidler et al. (2010) highlighted several factors that contribute to the age-related slowing of movements. Namely, changes in white matter (demyelination) (Zahr et al., 2009), gray matter (atrophy in the prefrontal cortex and also in the primary motor cortex) (Raz et al., 1997; Salat et al., 2004), and biochemical changes [decreased dopamine levels, receptors, transmission, and transporters (Gottfries, 1990; Kaasinen et al., 2000; Yamamoto et al., 2002)]. With respect to cortical motor cortex excitability, some studies suggest that motor cortex oscillations also depend on the inhibitory neurotransmitter γ-aminobutyric acid (GABA) (Gaetz et al., 2011; Burianová et al., 2020). Rossiter et al. (2014) reported that changes in beta oscillations in the motor cortex are associated with changes in GABA. Moreover, the meta-analysis by Porges et al. (2021) reported that GABA levels increase during adolescence and decrease later in adulthood. Thus, we can even speculate that the increased cortical excitability of the motor cortex may be due to the decreased GABA levels in older adults, which leads to decreased inhibitory processes. Further studies should clarify which processes (excitatory or inhibitory) play a greater role in the age-related changes in motor cortex excitability. It may be suggested that there is greater recruitment during planning and preparation of the motor response to the onset of a visual stimulus in the elderly compared to the younger participants. However, in the study by Yordanova et al. (2004), where only the upper limb was examined, similar findings were confirmed for more complex (choice) and not for simple RTs. Some discrepancies between the two studies were listed in the previous paragraph, but regarding MRCPs, our participants responded with the index finger, whereas Yordanova et al. (2004) used the middle finger. In our results, no such clear positivity was found at the ipsilateral site.

Although age-related slowing of simple RT tasks has been previously investigated using the EEG/ERP method for the upper-limb, this study extends knowledge to the lower-limbs. To be able to assess the fundamental responses to random time-locked visual stimuli, the simple RT task was applied. Previous study (Yordanova et al., 2004), however, showed that aging causes a functional dysregulation of the motor cortex that becomes more evident with increased task complexity. Although our study extends the information to the lower limbs, the fact that we do not have a paradigm of choice RT limits our understanding because some motor processes or mechanisms that might be associated with motor slowing in aging, such as response inhibition or mechanisms of limb selection, may not be revealed with the current paradigm. Thus, future studies should expand our protocol with simple RT with more complex cognitive tasks (Yordanova et al., 2004), with possible extension to the lower limbs. Randomizing responses for the upper- and lower limbs within the same block would lead to different results due to inhibitory processes of the brain regions representative of the hand and foot. Participants would have an equal chance of receiving visual stimuli requiring motor execution with the upper- or lower limb, representing a more complex paradigm. Finally, recent advances in Mobile Brain/Body Imaging (MoBI) research allow sensorimotor screening during movement (e.g., walking) in more naturalistic environments (Jungnickel and Gramann, 2016; Olsen et al., 2021; Wunderlich et al., 2021) would provide even more ecologically valid results.

In conclusion, our results to some extent confirm the previous findings showing an age-related slowing of sensorimotor activity occurring at the level of visual input as evidenced by visual-evoked potentials and motor response generation processes of the motor cortex, and further expand the results to the lower limbs. Quick and efficient reactions with upper- as well as lower-limb are needed to react to an unexpected hazard to avoid falls. The simple RT test evaluation is characterized as one of the basic measures that are highly dependent on sensorimotor integration and that estimates an individual’s alertness and processing speed. Our aim was to investigate the origin of age-related motor slowing at the neuronal level and to extend it to the lower limbs. Our study shows that age-related slowing already occurs during simple visual RT tasks with the upper and lower extremities and is not influenced by early processes of visual stimulus processing, but has its origin partly in the P2 component in addition to motor efficiency in the motor cortex. Because previous studies have shown that RTs can be improved with different physical (e.g., Fragala et al., 2014; Jehu et al., 2017) and cognitive (for review see Kueider et al., 2012) approaches, it would be important to extend this knowledge from the purely behavioral level to the neural level to explore which components are sensitive to training and to what extent they can be modulated. Such applications may be important not only for the aging population, but also for athletes who want to improve their performance to the millisecond.

## Data Availability Statement

The raw data supporting the conclusions of this article will be made available by the authors, without undue reservation.

## Ethics Statement

The studies involving human participants were reviewed and approved by the National Medical Ethics Committee (No. KME 57/06/17). The patients/participants provided their written informed consent to participate in this study.

## Author Contributions

UM, GD, BR, RP, and VK contributed to conception and design of the study. UM, MP, and NO contributed to data collection and organization of the database. UM, MP, KDP, NO, and VK contributed to data and statistical analysis. UM wrote the first draft of the manuscript. MP, KDP, and VK assisted with the interpretation of results. All authors contributed to manuscript revision, read, and approved the submitted version.

## Conflict of Interest

The authors declare that the research was conducted in the absence of any commercial or financial relationships that could be construed as a potential conflict of interest.

## Publisher’s Note

All claims expressed in this article are solely those of the authors and do not necessarily represent those of their affiliated organizations, or those of the publisher, the editors and the reviewers. Any product that may be evaluated in this article, or claim that may be made by its manufacturer, is not guaranteed or endorsed by the publisher.
