# Supplementary material for: Neural Bases of Age-Related Sensorimotor Slowing in the Upper and Lower Limbs
Source: Front Aging Neurosci. 2022 May 3;14:819576. doi: 10.3389/fnagi.2022.819576 (PMC9119024; doi:10.3389/fnagi.2022.819576)
Supplement: Supplementary file 1 [file Data_Sheet_1.docx]

Supplementary Material

## Supplementary Figures

**Supplementary Figure 1.** Stimulus-locked event related potentials (s-ERPs) for young (left) and old adults (right).


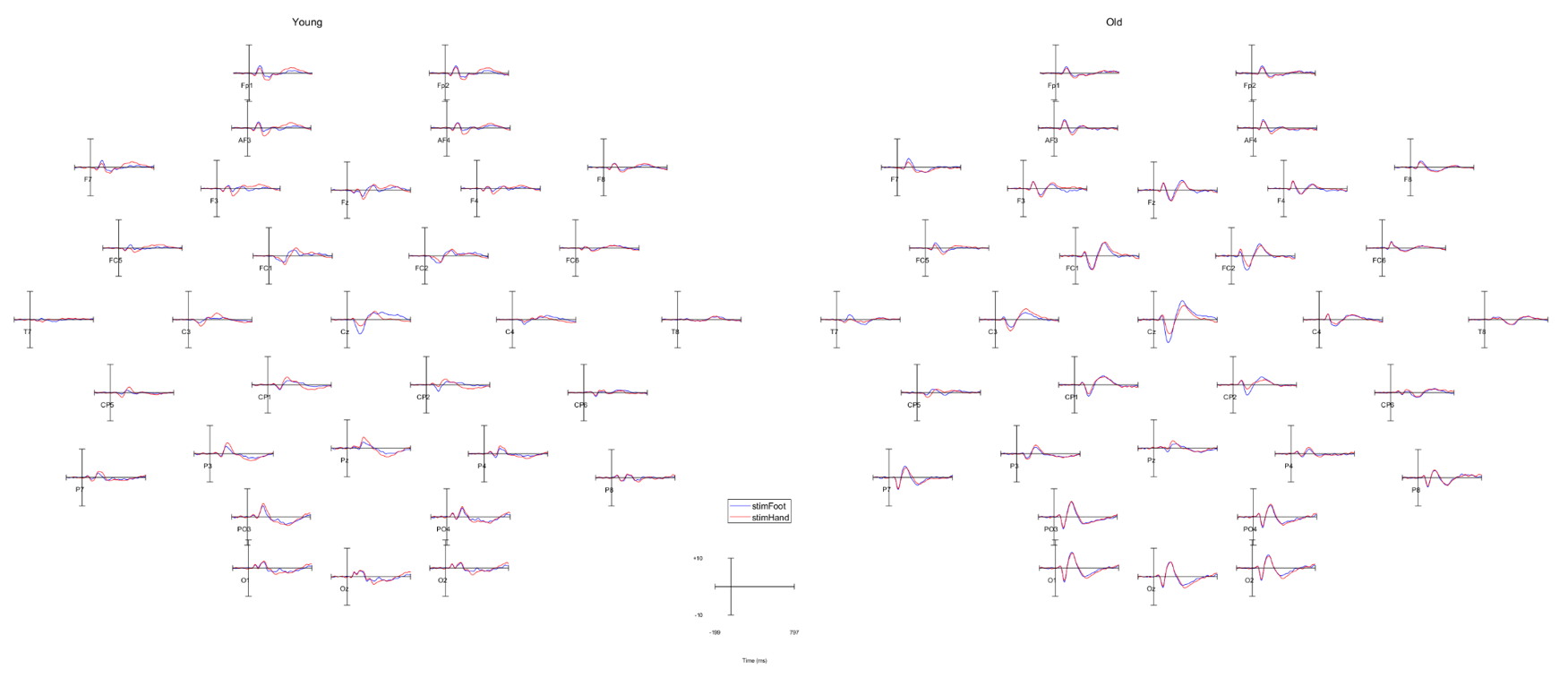


Note: Blue line represents the lower-limb RT, while red line represents the upper-limb RT.

**Supplementary Figure 2.** Response-locked event related potentials (r-ERPs) for young (left) and old adults (right).


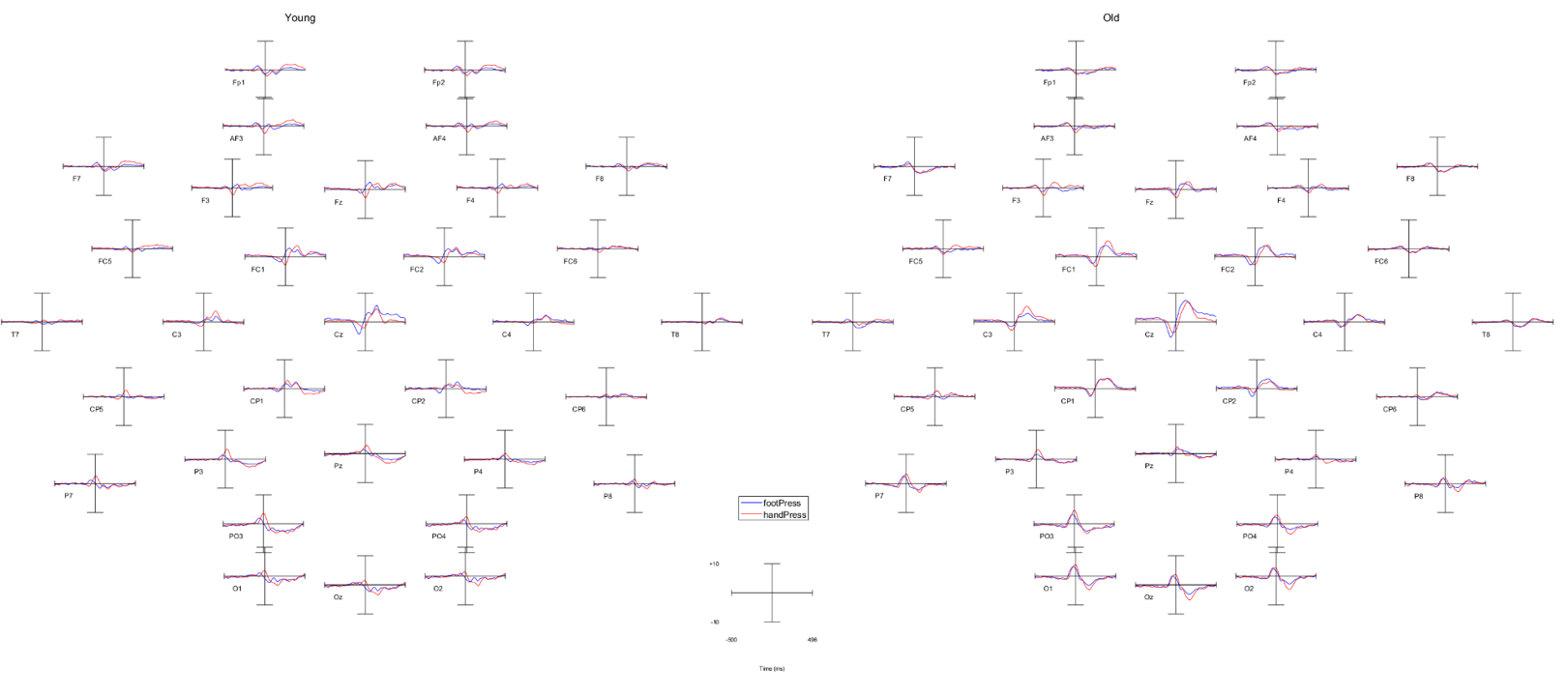


Note: Blue line represents the lower-limb RT, while red line represents the upper-limb RT.
